# Are there any *Pinus pinaster* trees resistant to *Bursaphelenchus xylophilus*? Studies implemented in Portugal to address this question

**DOI:** 10.1186/1753-6561-5-S7-P80

**Published:** 2011-09-13

**Authors:** Rita Costa, Patrícia Ribeiro, Isabel Evaristo, Bruno Ribeiro, Alexandre Aguiar, Isabel Carrasquinho, Carla Santos, Marta Vasconcelos

**Affiliations:** 1 Instituto Nacional dos Recursos Biológicos I.P. L-INIA – Quinta do Marquês, Av. da República, 2780-159 Oeiras, Portugal; 2 CBQF/Escola Superior de Biotecnologia, Universidade Católica Portuguesa, Rua Dr. António Bernardino de Almeida, 4200-072, Porto, Portugal

## 

The Pinewood nematode *Bursaphelenchus xylophilus* (PWN) was found to be responsible for maritime pine (*Pinus pinaster*) death for the first time in Europe in 1999, in a Portuguese forest south of Lisbon. It is the causal agent of the Pine Wilt Disease (PWD) and a quarantine organism in the European Union with approximately €80 million spent from 1999 – 2009 for attempting its eradication, which must also be added to direct losses from tree mortality. During this period, important progress has been made in understanding the relationships between PWN, its insect vector and the host tree/environmental factors that result in pine wilt in all countries affected but, especially, in Portugal.

Although PWN can cause devastating tree mortality, there is also evidence that some tree species or individual trees within a species are either tolerant (i.e. support the nematode, but are not killed immediately) or resistant to the nematodes. In order to investigate the susceptibility of *Pinus pinaster* to *Bursaphelenchus xylophilus* as a first step for the establishment of methodologies for the improvement of its resistance, a research program was implemented recently in Portugal for this species, combining genomic and quantitative genetics approaches. Until now, more than 500 trees were phenotypically selected in a highly affected stand and monitored during two years, regarding their phytossanitary status. The percentage of mortality observed in the first year was about 10%, with this value decreasing to less than 5% in the second year. Around 200 trees (including 150 of the selected trees) were genotyped for 6 microsatellite loci and three combinations of AFLP markers. The genotyping results show a high diversity of genotypes, which may open good prospects for the selection of resistant plants to *Bursaphelenchus xylophilus*.

At the same time, genomic and transcriptomic studies have been initiated with the final goal to discover candidate genes, loci and molecular markers related with disease resistance to PWN. With regards to the effects of the disease at a transcriptional level, the SSH technique was utilised to identify ESTs in *P. pinaster* and *P. pinea* when inoculated with PWN. ESTs were isolated, cloned, sequenced and identified using BlastN and BlastX, and clearly indicated that at an initial stage of the disease there is activation of a defence response at a molecular level, mainly related to oxidative stress, production of lignin and ethylene and post-transcriptional regulation of nucleic acids. 58% of the isolated sequences are not yet described, which shows the lack of genomic information currently available for pine. The results obtained are presented and discussed.

